# A Colony Multiplex Quantitative PCR-Based 3S3DBC Method and Variations of It for Screening DNA Libraries

**DOI:** 10.1371/journal.pone.0116997

**Published:** 2015-02-03

**Authors:** Yang An, Atsushi Toyoda, Chen Zhao, Asao Fujiyama, Kiyokazu Agata

**Affiliations:** 1 Department of Biophysics, Kyoto University, Kyoto, Kyoto, Japan; 2 Comparative Genomics Laboratory, National Institute of Genetics, Mishima, Shizuoka, Japan; 3 School of Pharmacy, Fudan University, Shanghai, China; University of Innsbruck, AUSTRIA

## Abstract

A DNA library is a collection of DNA fragments cloned into vectors and stored individually in host cells, and is a valuable resource for molecular cloning, gene physical mapping, and genome sequencing projects. To take the best advantage of a DNA library, a good screening method is needed. After describing pooling strategies and issues that should be considered in DNA library screening, here we report an efficient colony multiplex quantitative PCR-based 3-step, 3-dimension, and binary-code (3S3DBC) method we used to screen genes from a planarian genomic DNA fosmid library. This method requires only 3 rounds of PCR reactions and only around 6 hours to distinguish one or more desired clones from a large DNA library. According to the particular situations in different research labs, this method can be further modified and simplified to suit their requirements.

## Introduction

### Background

Although next-generation sequencing (NGS) is widely used at present, and has been used to assemble many genomes, DNA libraries still have irreplaceable roles. Firstly, by screening a DNA library, researchers can pick desired clones and get very precise sequences of specific genes within those clones. Secondly, even if a genome can be assembled from NGS data, there will still be gaps and uncertain DNA regions that need to be confirmed; screening a DNA library and sequencing targeted clones can help to achieve gap-closure and to evaluate and correct the assembled genome. Furthermore, assembly of some complicated genomes (with too many repetitive sequences or a high rate of heterozygosity or other variability) is extremely hard to accomplish by NGS alone, and therefore sequencing of DNA libraries is still usually an indispensable method for achieving whole-genome sequencing at present [1–2]. Thus, a DNA library is still a valuable resource for work such as molecular cloning, physical mapping of genes, and comparative genomics.

To take the best advantage of DNA libraries, a large number of library screening methods have been developed during the past few decades [3–4]. In early studies, library screening was mainly based on hybridization between clones containing recombinant DNA vectors (bacteriophage, cosmid, plasmid or fosmid), and specific probes (radioactive or synthetic oligonucleotide probes) [5–12]. Later, to avoid the low signal-to-noise ratio and considerable cost of hybridization, PCR-based screening methods were developed. In PCR-based methods, one first isolates DNA from pools of clones, and then uses primers designed to screen the positive pools containing desired clones by PCR, and finally identifies the positive clones by hybridization or further PCR reactions [13–20]. The development of colony PCR [21–23] and quantitative PCR (qPCR) [24] made this method much easier to perform.

However, despite the advantages of the PCR-based screening method, it is still not efficient enough for some requirements of modern genomic research. The arbitrary, inefficient pooling strategy, the culturing of clones, DNA extraction, numerous PCR steps and electrophoresis procedures generally used for PCR-based screening are time-, money-, and labor-consuming and produce many false-positive results. In order to take better advantage of DNA libraries, here we describe an efficient colony multiplex quantitative PCR-based 3–step, 3-dimension, and binary-code (3S3DBC) method for screening of DNA libraries.

### Mathematics of DNA library screening

Screening out one desired clone from a DNA library can be considered a mathematical problem, i.e., how to distinguish one positive sample among a large number of samples. A good library screening method means an optimal solution that needs the least time (i.e., few detection steps, e.g., few PCR rounds in the case of PCR-based screening) and least labor (i.e., simple pooling procedure and small detection number, e.g., a small number of PCR reactions needed in the case of PCR-based screening) to solve this mathematical problem. Usually, screening uses one of three different methods: the dimension-based method, bisection-based method or binary code-based method.

**Dimension-based method**. Dimension-based methods have been widely used for screening. A one-dimensional method means that all samples are aligned in a one-dimensional line, and the desired sample can be detected by screening them one by one (S1 Fig.). In a two-dimensional method, all samples are arranged into a two-dimensional square (S2 Fig.). After pooling the samples of each row and column, and screening these pools, the desired sample is identified as occupying the intersection of the positive row and column. A three-dimensional method means that all samples are arranged into a three-dimensional cube (S3 Fig.). After pooling the samples of each layer in the three-dimensional cube and screening them, the desired sample is the sample located at the intersection of the three positive layers. Similarly, in a four-dimensional method, all samples can be arranged into a four-dimensional hypercube, and after pooling the samples of the cubes in each dimension and screening them, the desired sample is located at the intersection of the positive cubes. Furthermore, samples can also be arranged into five-, six-, and so on, dimensions. As it is nearly impossible to depict arrangements in more than three dimensions in our three-dimensional world, some variants of a high-dimensional method can be used for pooling (S4 and S5 Figs.). The lowest detection number “n” of the dimension-based method equals $n=D*\sqrt[{\text{D}}]{\text{N}}$ (D is the dimension number and N is the total number of samples). A higher dimension method seems better because the higher the dimension number, the lower the detection number required; however, at the same time, it requires more complicated procedures for pooling samples.

**Bisection-based method**. Another simple and commonly used method for screening out one sample from a large sample set is the bisection-based method (S6 Fig.). This method requires several detection steps. In each step, the sample universe is divided into two equal subsets (pools of samples), and the positive subset is detected. The division and detection procedure is repeated at each step until only one sample, which is the desired sample, is left in the final positive subset. The number of detection steps, “n”, of this method is n = 2*log_2_^N^-1 (N is the total number of samples). This exponential bisection method only needs a small number of detections; however, it needs many detection steps (and thus long working time), because the detection at each step is based on the result of the previous detection step.

**Binary code-based method**. In the binary code-based method (S7 Fig.), at first, all samples’ assigned decimal code numbers are aligned in a column. Then, all of these decimal code numbers can be converted to binary code numbers, which are arranged in a matrix. In this binary code number matrix, for each column, samples whose assigned binary code numbers include the digit 1 are mixed to form a pool. After detection, each positive pool is marked “1”, and each negative pool is marked “0”. The final binary code can be converted back into a decimal code that indicates the position of the real positive sample. The detection number (n) equals the total number of pools, n = log_2_^N^ (N is the total number of samples). This method uses a smaller number of detections compared with the other methods, and all detections can be performed simultaneously and independently. For instance, to screen out one specific sample from 262144 samples requires 193 detections by the 3-dimensional method, 35 detections by the bisection-based method, but only 18 detections by the binary code-based method.

### Issues that must be considered when designing the practical details of the screening method

Although several mathematical solutions have thus been provided for screening out one desired sample from a sample universe, real cases are much more complicated than the simple mathematical problem. In our actual case, for example, our *Dugesia japonica* planarian DNA fosmid library contained 161,280 clones, which equaled 4-fold coverage of one planarian genome-equivalent. Therefore, a single copy gene could theoretically have 4 possible clones in this library. How to decrease the screening time, labor and cost, but increase the screening sensitivity and accuracy, is always the goal in designing any screening experiment. Accordingly, issues of the detection method and the pooling strategy of the screening design and some other practical considerations are discussed next (S1 Table).

**Detection method**. The detection method generally used for DNA library screening has changed over time from hybridization to PCR. Although the PCR method has the advantage of high sensitivity, the clone culturing and DNA extraction before PCR, and the large number of PCR rounds and electrophoresis procedures, are still time- and labor-consuming, which makes the library screening tedious work. In this study, we used a colony multiplex quantitative PCR detection method to make the library screening less tedious. Quantitative PCR (qPCR) can use stored clones directly, and the detection is based only on checking the qPCR dissociation curve without requiring culturing of clones, DNA extraction, or electrophoresis steps. Multiplex primer sets can make it possible to screen several genes simultaneously (different qPCR products can be distinguished by their unique peak position in the dissociation curve). Using more primer sets can save time, labor and money by reducing the number of PCR reactions and PCR rounds in the screening; however, it also decreases the detection sensitivity and screening accuracy, since too many primer sets will disturb the PCR reaction and the ability to distinguish among PCR products.

**Pooling density and PCR detection sensitivity**. Pooling is used in nearly all PCR-based library screening; and pooling density is one of the most crucial issues that need to be considered because it is related to PCR sensitivity and accuracy, and the number of PCR reactions and rounds, which in turn affect the time, labor and money used for screening. For the dimension-based method, two-dimensional (row/column pools) [17,25] and three-dimensional (plate/row/column pools) pooling [18–20,23–24,26–28] strategies have commonly been used. Various higher-dimensional pooling strategies [10,15,29–30] have also been described. Higher-dimensional pooling helps to decrease the number of PCR reactions necessary during screening, and thus to decrease the time, money and labor consumed. However, higher-dimensional pooling is also computationally complicated, and the pooling itself is labor-consuming if appropriate automation machinery is not available. At the same time, higher-dimensional pooling means higher pooling density, which can lead to the concentration of an individual target sample becoming too low to be detected by PCR. Similarly, a bisection-based method may not be suitable for practical screening work, because, in the initial pooling step, this method needs to separate all samples into two super pools, which may result in such high pooling density that the sensitivity of PCR detection is not high enough. To use a section-based method, some studies tried to decrease the pooling density by separating clones into a larger number of super pools [16]. The binary code-based method has a similar problem. This method has been used for detecting protein-protein interaction [31]; however, no such method has been described for DNA library screening. Overly high pooling density is one crucial problem that would impede its application. So, considering the PCR detection sensitivity, a suitable pooling density is important for a PCR-based screening method. To test the PCR detection sensitivity in our planarian DNA library screening, we made and screened some pools with an increasing number of clones. PCR and qPCR experiments showed that PCR was not sufficiently sensitive to detect one sample from a super pool containing a mixture of 10^4^ or more clones.

**Detection accuracy and false positive problem**. In a practical case, if a DNA library covers more than one genome-equivalent, the false-positive issue should be taken into account. More positive samples induce more false-positive results in dimension-based and binary code-based methods, and increase the detection number in section-based methods (S8 Fig.). In dimension-based methods, the largest possible number of false-positive results (N) equals n^D^-n (where n is the number of true-positive results, and D is the number of dimensions) (S9 Fig.). As the number of dimensions increases, more false-positive results appear, which results in low PCR detection accuracy. The case is much worse for binary code-based methods, because the binary code method can only detect one unique sample from a pool. If there is more than one positive sample in the same pool, the binary code method will give a wrong result that will seriously affect the screening accuracy (S10 Fig.). So, in order to avoid false-positive results, the higher the probability that one super pool contains only one desired clone after pooling, the better. In theory, one single copy gene has 4 positive clones in our planarian DNA library, so the probability (P) that one positive clone will appear in only one super pool equals $P=1*\frac{n-1}{n}*\frac{n-2}{n}*\frac{n-3}{n}$ (n is the number of super pools). The tradeoff between P and n should thus be carefully balanced.

**Automation**. It is obvious that as the pooling density increases, manual pooling will become a much more complicated challenge, and a labor- and time-consuming task that would probably be impossible to accomplish. This point appears to be one of the main limitations of the high-density pooling strategy. Therefore, the use of a suitable robot should be very important for optimizing the pooling strategy, simplifying screening procedures and reducing cost. In our actual case, our robot could only make one clonal pool from each 384-well plate. So, because of the robot performance limitation, we used two steps to separate a super pool into 3 dimensions (one step for plate dimension and one step for row and column dimensions).

## Results

### Screening out a gene by the colony multiplex quantitative PCR-based 3S3DBC DNA screening method

**Figure 1:**
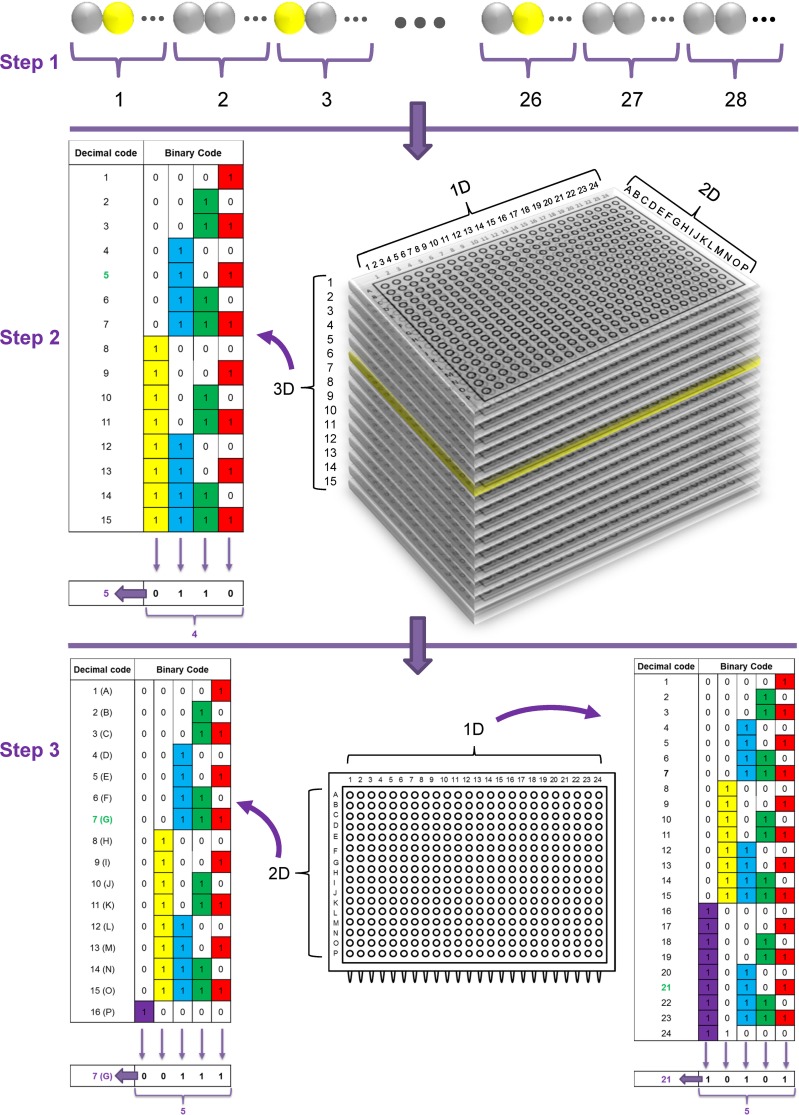
A colony multiplex quantitative PCR-based 3S3DBC DNA screening method for planarian DNA library screening.

Based on the issues described above, the final solution we devised for our *Dugesia japonica* fosmid library screening work was a “colony multiplex quantitative PCR-based 3S3DBC screening strategy” (Fig. 1). We used the known sequence from clone DJF-033N19 (this name indicates that the location of this sequence in our *Dugesia japonica* fosmid library was Plate No. 033, Row No. N, Column No. 19) as a positive control, and DjPiwiB was the desired gene we wanted to screen out from this library. Our strategy needed only 3 steps to identify one desired clone from our whole library.

**Figure 2:**
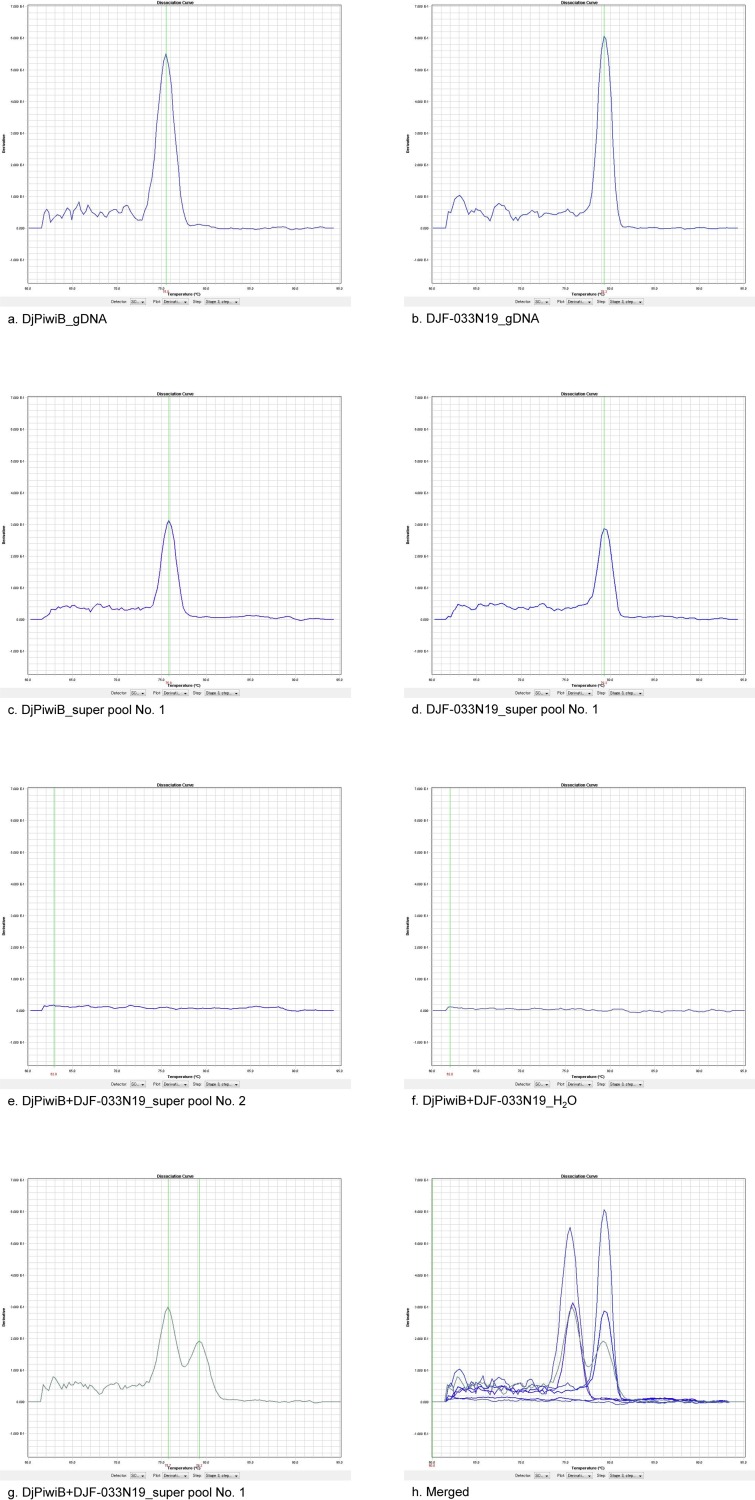
Representative results of qPCR disassociation curves during screening. This figure was snipped from our results obtained using ABI qPCR analysis software SDS 2.4 standalone, and shows representative results of disassociation curves generated from the first step of 3S3DBC DNA screening. Positive DNA template (10 ng of *Dugesia japonica* genomic DNA) was used to detect the qPCR specificity of the respective primer set of DjPiwiB (a) and DJF-033N19 (b). Each primer set could also generate the specific qPCR product after qPCR in a real library super pool (c and d). No nonspecific amplification was detected in the negative super pool or in the H_2_O negative template control, using either DjPiwiB or DJF-033N19 multiplex primer sets (e and f). The expected qPCR disassociation curve (double peak shows two qPCR products) was detected in the positive library super pool using DjPiwiB and DJF-033N19 multiplex primer sets (g), and the dissociation temperature values of the two peaks corresponded to the same values obtained in the positive template control and positive super pool results. All figures from (a) to (g) were merged to produce (h).

In the first step, in order to make the probability at least 80% that our desired clone appeared no more than once in a super pool, we mixed a total of 161280 clones into 28 super pools. Each super pool was constructed by using fifteen 384-well plates. We chose the number fifteen because it is 2^n^-1 (n is a natural number), which could be efficiently used in the following binary code configuration. The total mixed clone number in one super pool was 5760, which was small enough (less than 10^4^) for qPCR detection sensitivity. After the detection of 28 simultaneous qPCR reactions, positive super pools were found as verified by qPCR disassociation curves (Fig. 2); moreover, this experiment verified that multiplex primer sets can be successfully used to screen multiple genes simultaneously with this method. The positive super pools for the positive control sequence DJF-033N19 were super pool No. 1 (384-well plates No. 1 to No. 15) and super pool No. 3 (384-well plates No. 31 to No. 45), and the positive super pools for the DjPiwiB gene were super pool No. 1 and super pool No. 2 (384-well plates No. 16 to No. 30).

In the second step, the positive cuboid super pool was pooled by its third dimension—the plate layer dimension. Then, the binary-code pooling method was further used to obtain much higher pooling density. A total of 4 simultaneous qPCR reactions were sufficient to screen out one positive plate from 15 plates. From this step, the positive plates for the control gene clone DJF-033N19 were plate No. 004 and plate No. 033, which indicated that we found the correct positive plate and one extra positive plate. Also for the DjPiwiB gene, we found two positive plates (No. 006 and 025).

In the third step, each positive plate was further pooled into a row dimension pool and a column dimension pool. The binary-code pooling method was again used to make higher pool density of the pools for each dimension. Only 5 simultaneous qPCR reactions were needed to screen out the positive row or column, and to finally distinguish the desired clone from the intersection between the positive row and column. We finally found two positive clones (DJF-033N19 and DJF-004C08) for the positive control DJF-033N19, and two positive clones (DJF-006G21 and DJF-025C08) for the DjPiwiB gene. The positive control demonstrated that we obtained a correct screening result using this method, and later sequencing of the two DjPiwiB gene-positive clones also demonstrated the accuracy of this screening result. Accordingly, in the case of our planarian fosmid library, this colony multiplex quantitative PCR-based 3S3DBC screening method only needed 42 qPCR reactions and less than 7 hours to screen out one desired clone from a DNA library containing 161,280 clones.

## Discussion

Here we first described general considerations regarding the detection method and pooling strategy for screening a DNA library, and then detailed our rapid and low-cost new screening method—a colony multiplex quantitative 3S3DBC method, which is superior to the conventional 3D screening method (S2 Table). By considering the time, labor, cost, detection sensitivity, detection accuracy, automation and other issues in a particular DNA library screening, this method can be modified to produce several other variant methods. For example, if a DNA library has very few clones which altogether cover less than one genome-equivalent, and if a suitable automation robot is available to perform complicated pooling, a 1-step binary code screening method might be adequate. However, usually a DNA library contains hundreds of thousands of clones that cover more than 1 genome-equivalent, so an additional pooling step should be added to make more super pools in order to decrease pooling density and to increase the PCR detection sensitivity and the probability that one positive clone will appear no more than once in a super pool (2-step binary code pooling). Dimension-based pooling is simpler than binary code-based pooling, if a pooling robot is not available. Increasing the number of PCR steps can also help to reduce pooling complications. In addition, since overly high pooling density can reduce PCR detection sensitivity, more pools are required, or a pre-PCR step before pooling is an alternative method to increase PCR detection sensitivity [24]. We also showed here that multiplex qPCR can be used to screen multiple genes simultaneously (we used two primer sets in our experiment). Presumably, more genes could be screened simultaneously by using more than 2 primer sets, but of course, the number that could be screened would be limited by the same factors (i.e., primer sets design, rate at which primers anneal to their targets, buffer constituents, and annealing temperature, which affect PCR sensitivity and specificity) that limit any multiplex PCR method [32–34]. In conclusion, by making trade-offs and using flexible combinations of approaches to address these issues, our 3S3DBC DNA library screening method can be modified and widely used for screening a variety of DNA libraries, and further employed for other screening-like experiments such as protein-protein interaction and hybridization tests.

## Materials and Methods

### *Dugesia japonica* planarian genomic DNA library

The DNA library was constructed by the National Institute of Genetics in Tokyo Japan. Colonies were picked into freezing solution that contained ampicillin (50 ug/ml), and stored at −80°C.

### Pooling

Clones (4 ul/clone) from each 384-well plate were mixed into a plate pool using a BioTech EDR-384S II Multi-functional Table Top Pipette Station. Row- and column-pools from each plate were made manually using Eppendorf multichannel pipettes. Each 15-plate pool was further mixed to produce a super pool.

### qPCR kit and conditions

The QuantiTect SYBR Green PCR kit was used. According to the manufacturer’s qPCR reaction kit instructions, sometimes adding a small amount of extra ExTaq enzyme (0.05ul/10ul reaction) will yield a better result. The qPCR reactions were performed on an ABI PRISM 7900HT Sequence Detection System, and the qPCR cycling conditions were: 95°C for 10 mins, [95°C for 30 seconds, 57°C for 30 seconds, 72°C for 50 seconds] (40 cycles), 72°C for 7 mins, followed by dissociation curve analysis.

### Primers for DjPiwiB gene and positive control gene

The DjPiwiB primer set was DjPiwiB_Fw (5′-ATGGATCCCATGGCTCCTAATG-3′) and DjPiwiB_Rv (5′-TGCACAGGGACAGGTACACG-3′). The clone DJF-033N19 (plate No.33, row N and column No. 19) was sequenced previously. Its location and known sequence were used for a positive gene control. The primer set for this sequence was DJF-033N19_Fw (5′-AATCGGGAGAACGGGAAGATGTG-3′) and DJF-033N19_Rv (5′-GCCATTCGGAACTTGAGCTTGAC-3′).

## Supporting Information

S1 Fig One dimension-based method. One-dimensional method means that all samples are aligned in a one-dimensional line, and the desired sample can be detected by screening them one by one.
(TIF)

S2 Fig Two dimension-based method. In a two-dimensional method, all samples are arranged into a two-dimensional square. After pooling the samples of each row and column, and screening these pools, the desired sample is identified as occupying the intersection of the positive row and column.
(TIF)

S3 Fig Three dimension-based method. A three-dimensional method means that all samples are arranged into a three-dimensional cube. After pooling the samples of each layer in the three-dimensional cube and screening them, the desired sample is the sample located at the intersection of the three positive layers.
(TIF)

S4 Fig Six dimension-based method. (TIF)

S5 Fig Nine dimension-based method. (TIF)

S6 Fig Bisection-based method. Bisection-based method requires several detection steps. In each step, the sample universe is divided into two equal subsets (pools of samples), and the positive subset is detected. The division and detection procedure is repeated at each step until only one sample, which is the desired sample, is left in the final positive subset.
(TIF)

S7 Fig Binary code-based method. In this binary code number matrix, for each column, samples whose assigned binary code numbers include the digit 1 are mixed to form a pool. After detection, each positive pool is marked “1”, and each negative pool is marked “0”. The final binary code can be converted back into a decimal code that indicates the position of the real positive clone.
(TIF)

S8 Fig Problem of section-based method when multiple copies of the desired sample are located in the same pool. Although the section-based method can give the correct result, it requires many more detection numbers
(TIF)

S9 Fig Problem of dimension-based method when multiple copies of the desired sample are located in the same pool. False-positive results are a common issue when more than one desired sample exists in the same pool. For the dimension-based method, when the dimension number is greater than 1, the largest number of false-positive results “N” equals n^D^-n (D is the dimension number and n is the number of true positive samples in the library).
(TIF)

S10 Fig Problems of binary code-based method when multiple copies of the desired sample are located in the same pool. The binary-code method even produces a wrong result in this case (for example, if samples No. 2 and No. 5 are both positive samples, this method will give a wrong result: No. 7).
(TIF)

S1 Table Relationships among issues that must be considered when designing a screening method. Positive and negative relationships of issues (x, y and z) that must be considered when designing a screening method are listed in the table. The functions relating x, y and z (z = f(y); y = f(x)), and their suitable solutions depend on different research laboratories’ particular situations.
(DOCX)

S2 Table Comparison of screening one desired clone from DNA library by 3S3DBC screening method and conventional 3-dimensional method. (DOCX)
