# Supplementary figures and images for: A Colony Multiplex Quantitative PCR-Based 3S3DBC Method and Variations of It for Screening DNA Libraries
Source: PLoS One. 2015 Feb 3;10(2):e0116997. doi: 10.1371/journal.pone.0116997 (PMC4315571; doi:10.1371/journal.pone.0116997)

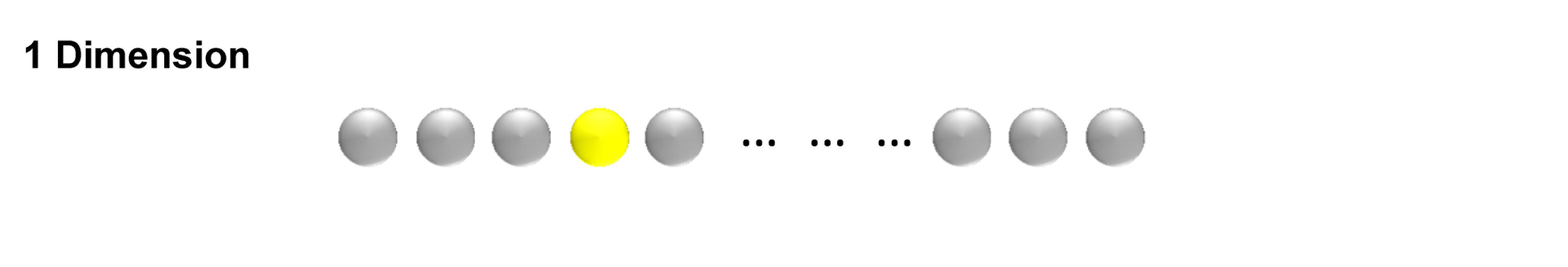

Supplement: S1 Fig — One-dimensional method means that all samples are aligned in a one-dimensional line, and the desired sample can be detected by screening them one by one. (TIF) [file pone.0116997.s001.tif]

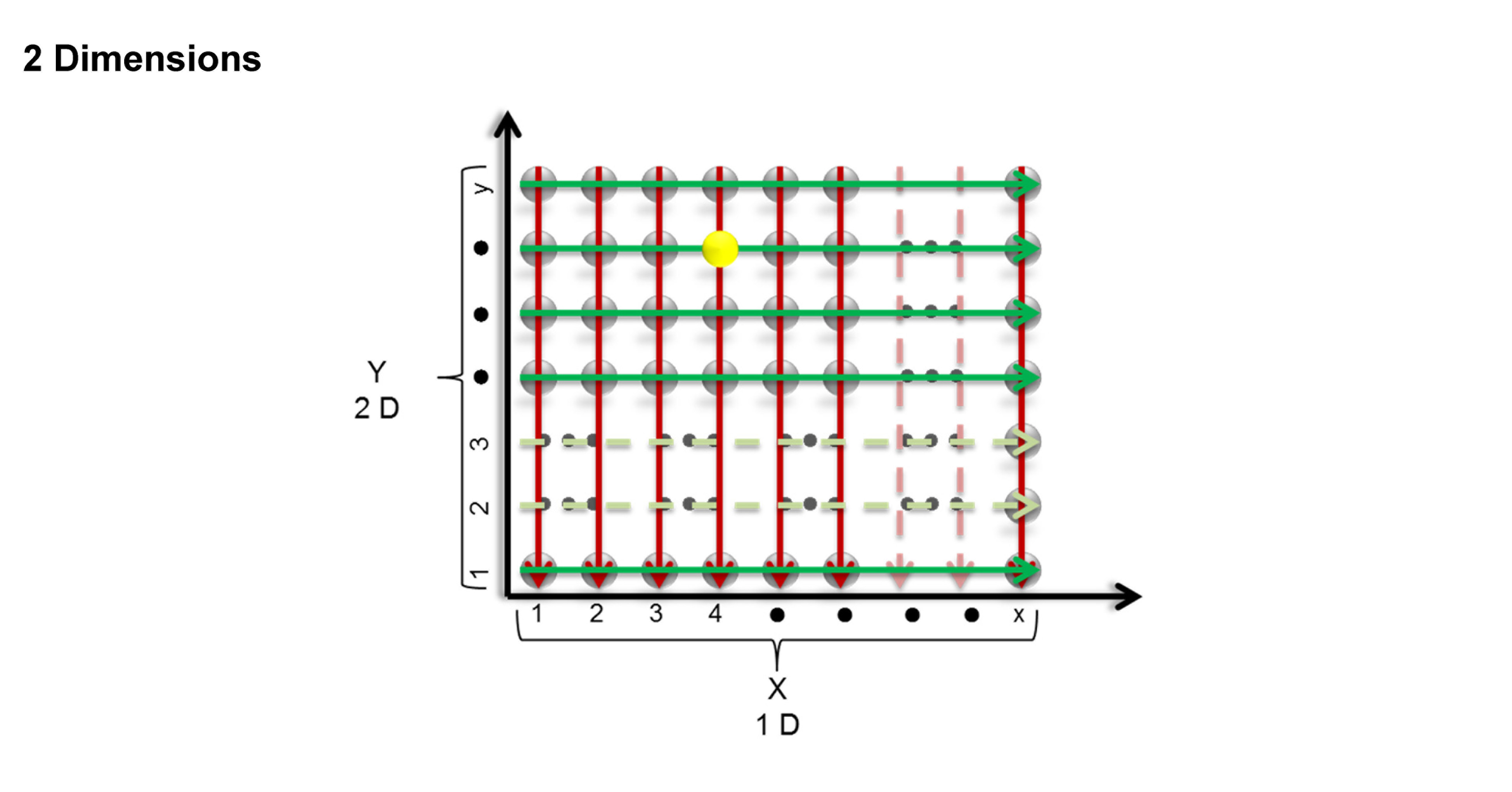

Supplement: S2 Fig — In a two-dimensional method, all samples are arranged into a two-dimensional square. After pooling the samples of each row and column, and screening these pools, the desired sample is identified as occupying the intersection of the positive row and column. (TIF) [file pone.0116997.s002.tif]

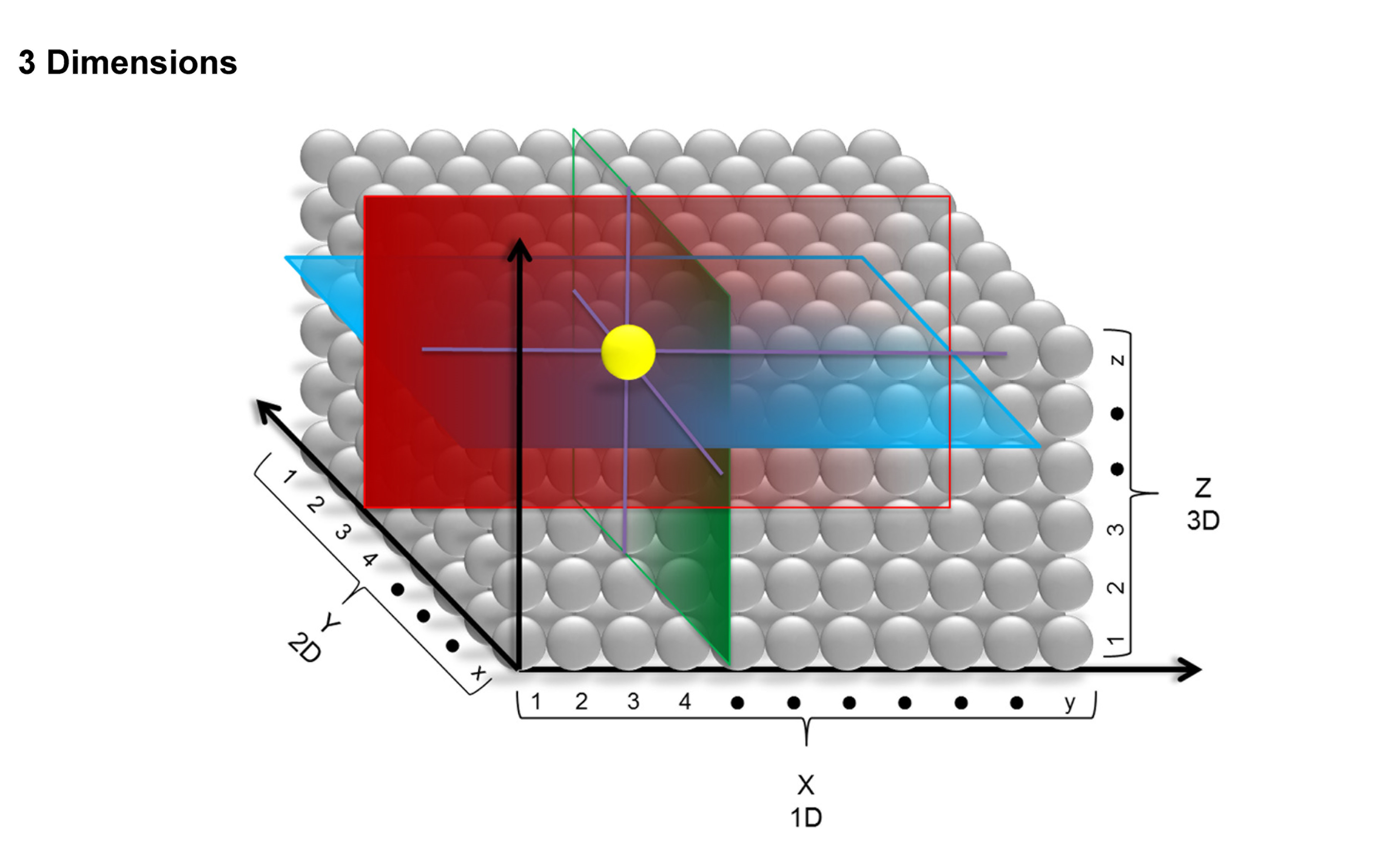

Supplement: S3 Fig — A three-dimensional method means that all samples are arranged into a three-dimensional cube. After pooling the samples of each layer in the three-dimensional cube and screening them, the desired sample is the sample located at the intersection of the three positive layers. (TIF) [file pone.0116997.s003.tif]

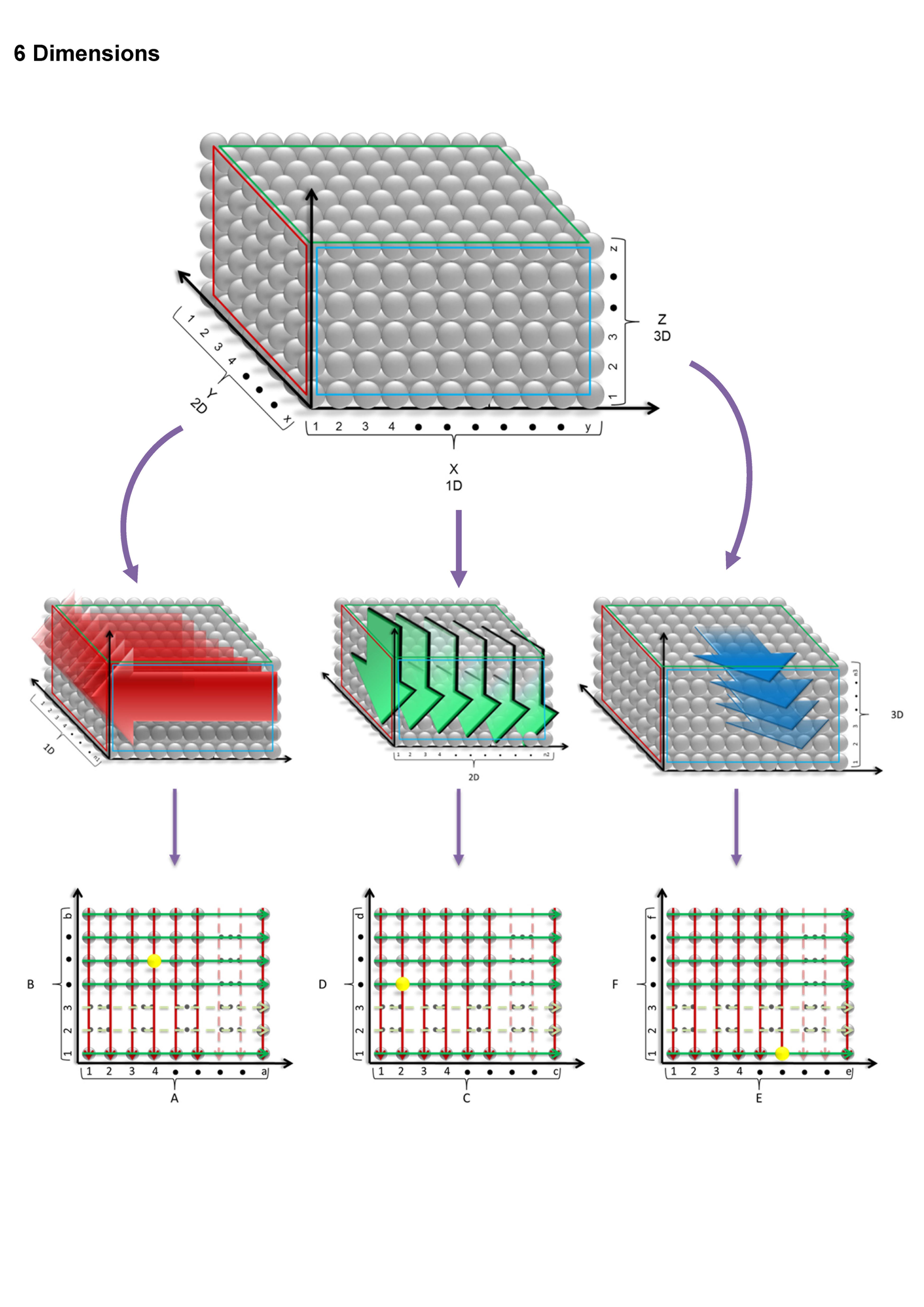

Supplement: S4 Fig — (TIF) [file pone.0116997.s004.tif]

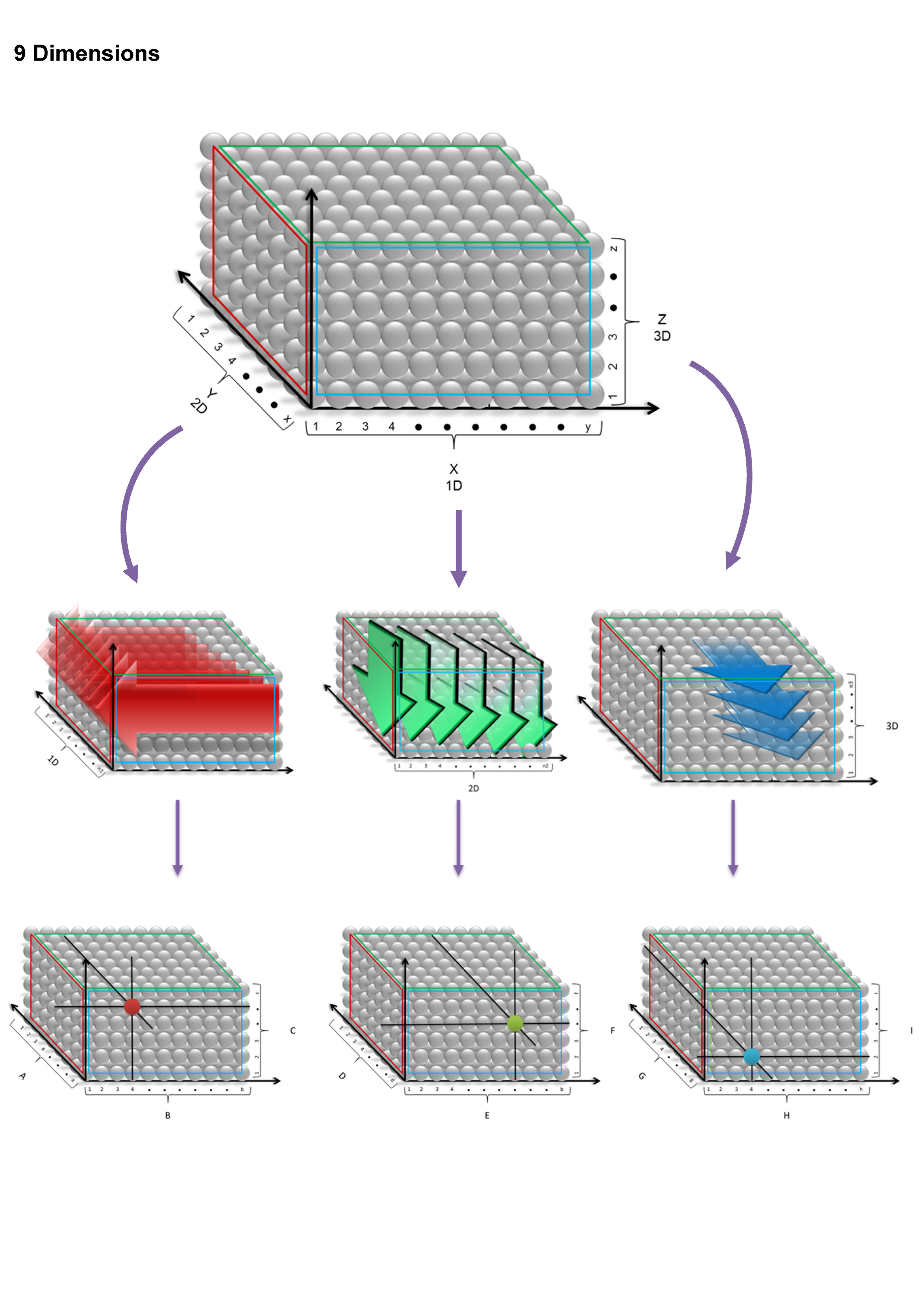

Supplement: S5 Fig — (TIF) [file pone.0116997.s005.tif]

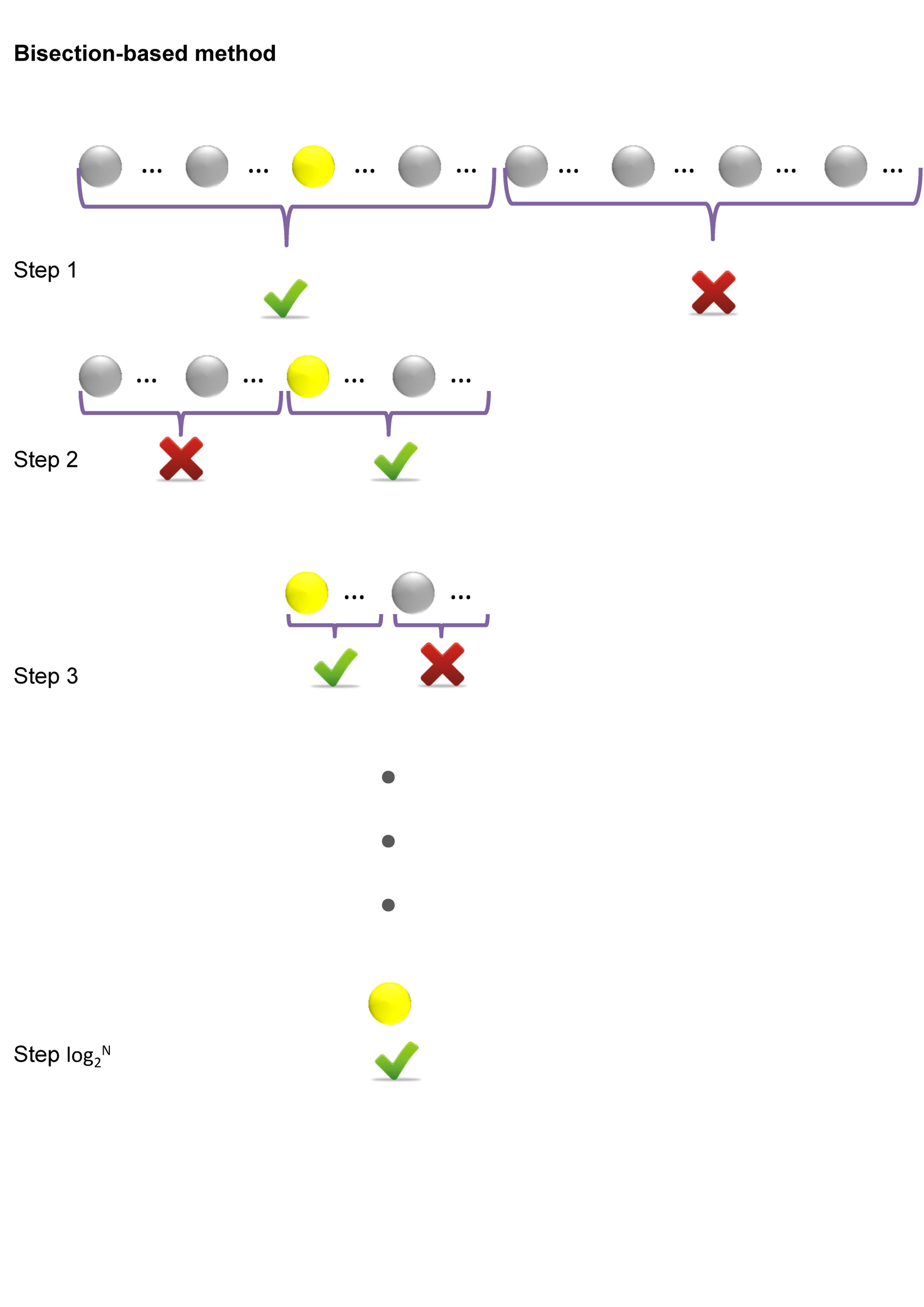

Supplement: S6 Fig — Bisection-based method requires several detection steps. In each step, the sample universe is divided into two equal subsets (pools of samples), and the positive subset is detected. The division and detection procedure is repeated at each step until only one sample, which is the desired sample, is left in the final positive subset. (TIF) [file pone.0116997.s006.tif]

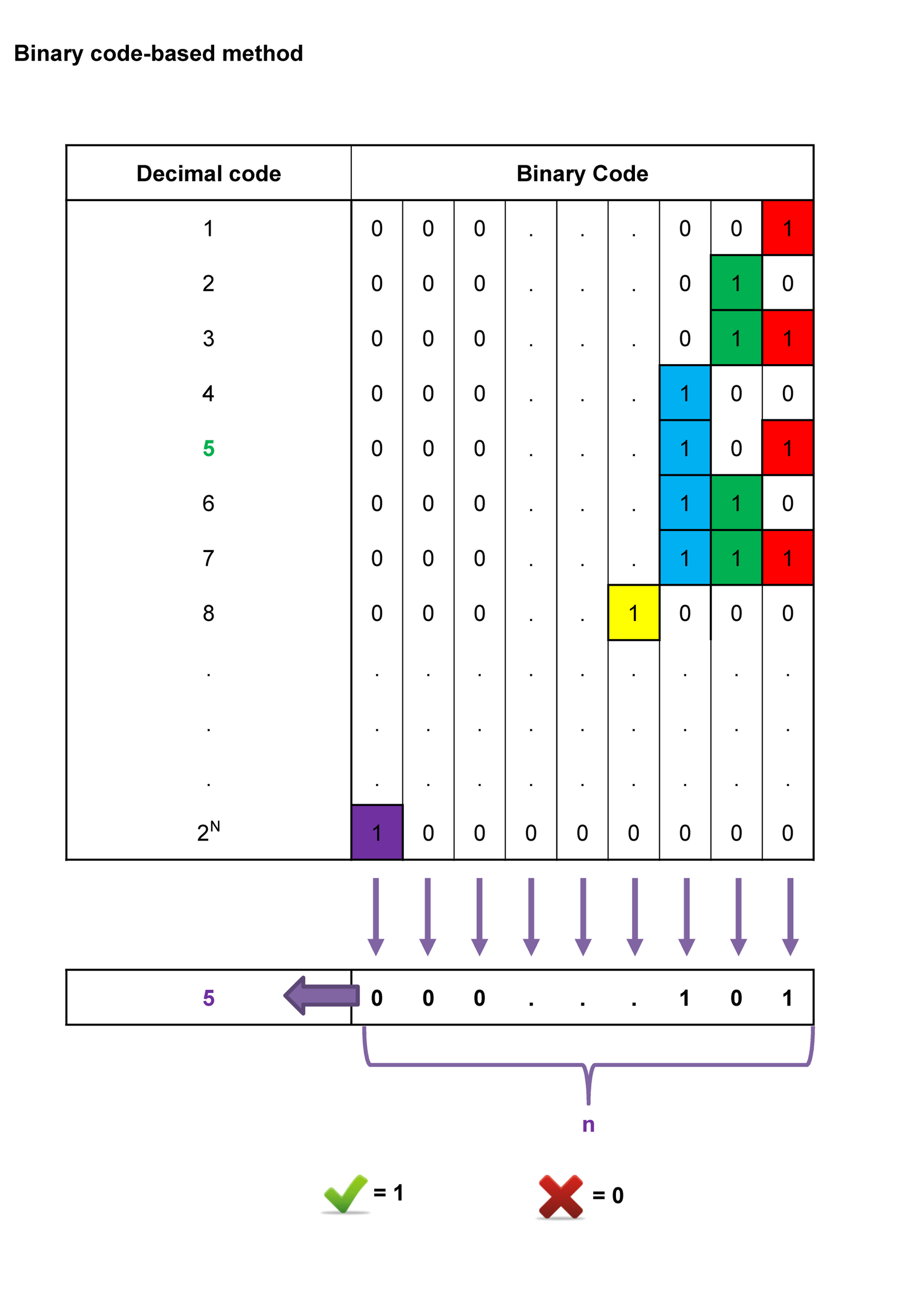

Supplement: S7 Fig — In this binary code number matrix, for each column, samples whose assigned binary code numbers include the digit 1 are mixed to form a pool. After detection, each positive pool is marked “1”, and each negative pool is marked “0”. The final binary code can be converted back into a decimal code that indicates the position of the real positive clone. (TIF) [file pone.0116997.s007.tif]

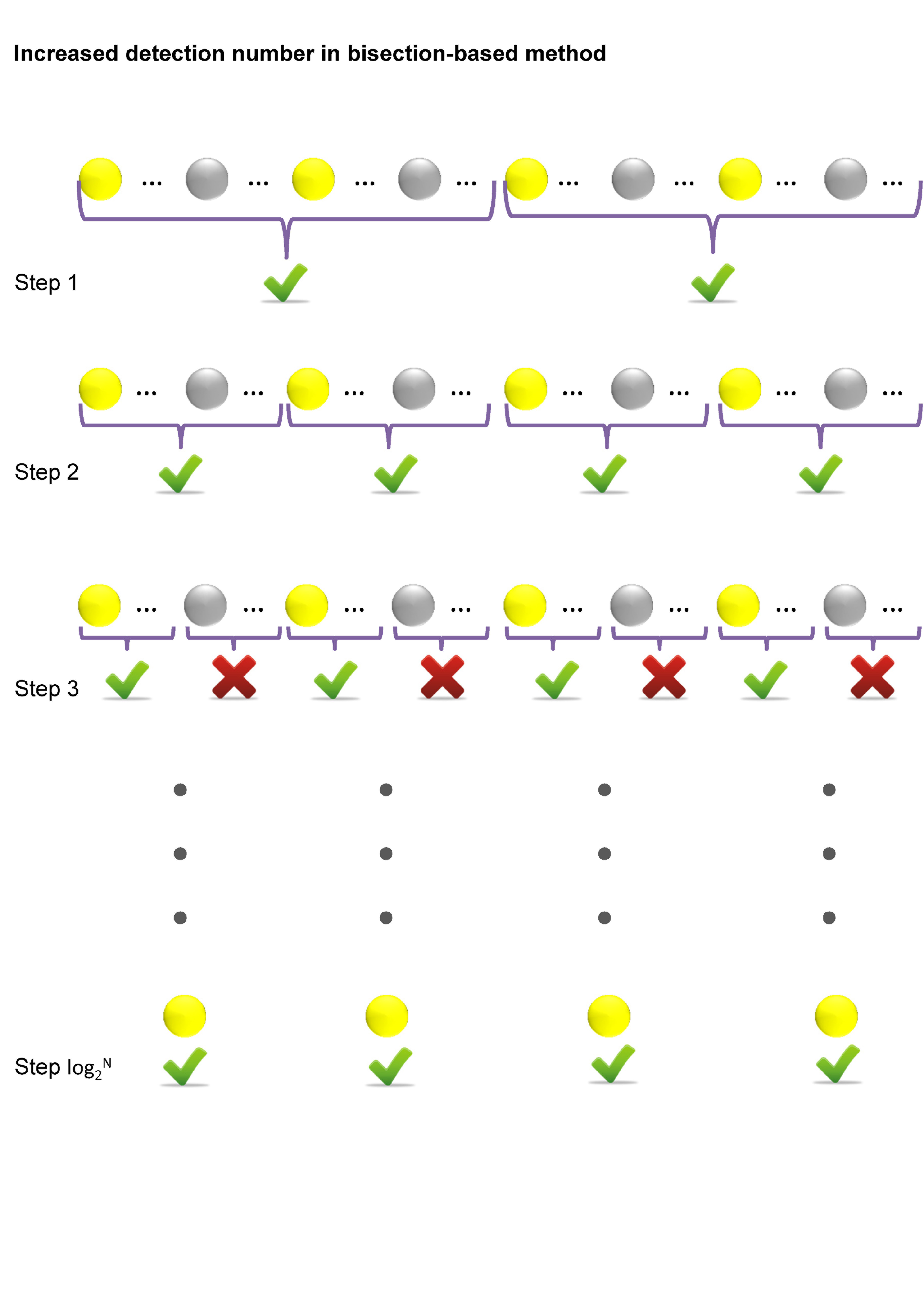

Supplement: S8 Fig — Although the section-based method can give the correct result, it requires many more detection numbers (TIF) [file pone.0116997.s008.tif]

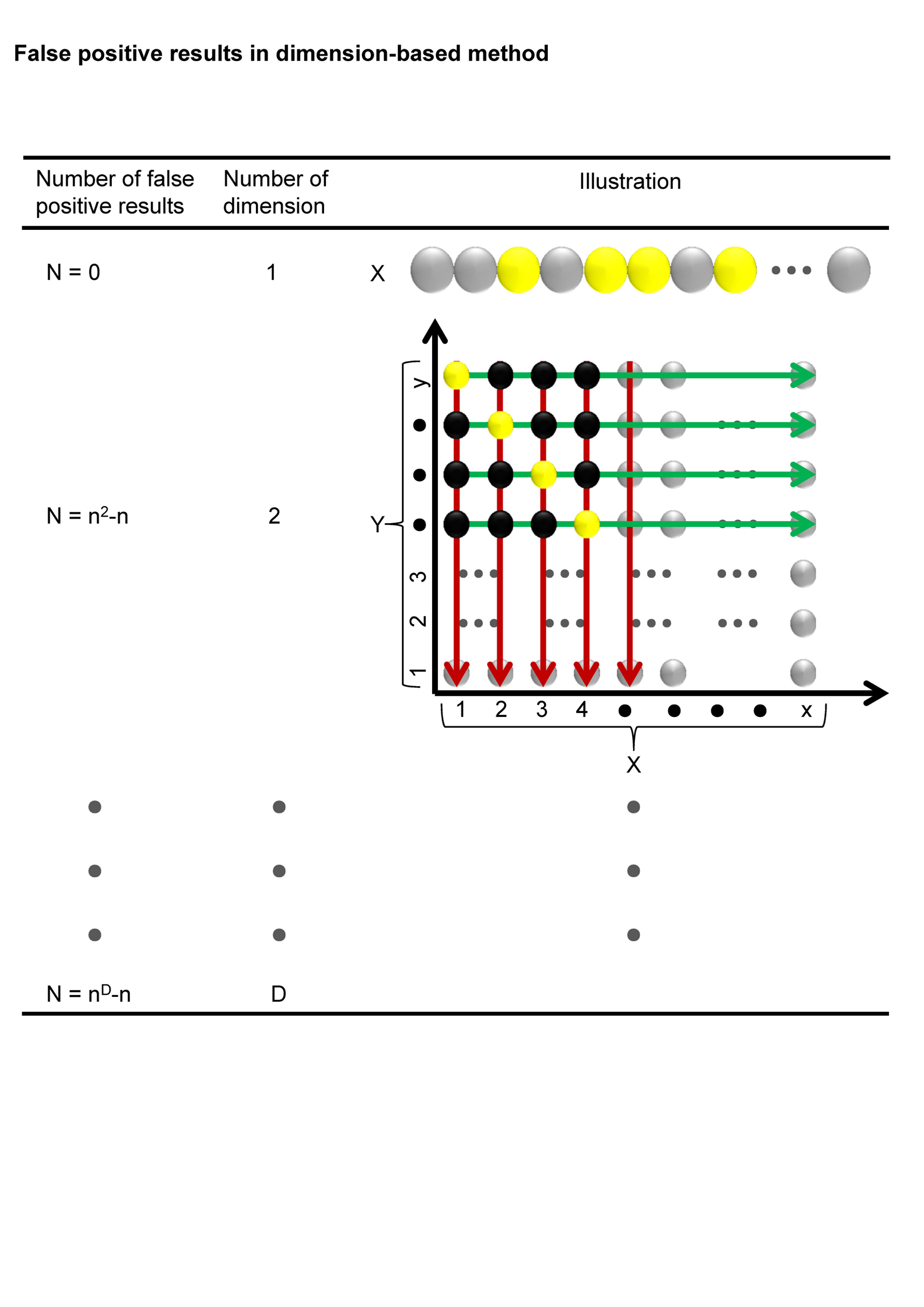

Supplement: S9 Fig — False-positive results are a common issue when more than one desired sample exists in the same pool. For the dimension-based method, when the dimension number is greater than 1, the largest number of false-positive results “N” equals nD-n (D is the dimension number and n is the number of true positive samples in the library). (TIF) [file pone.0116997.s009.tif]

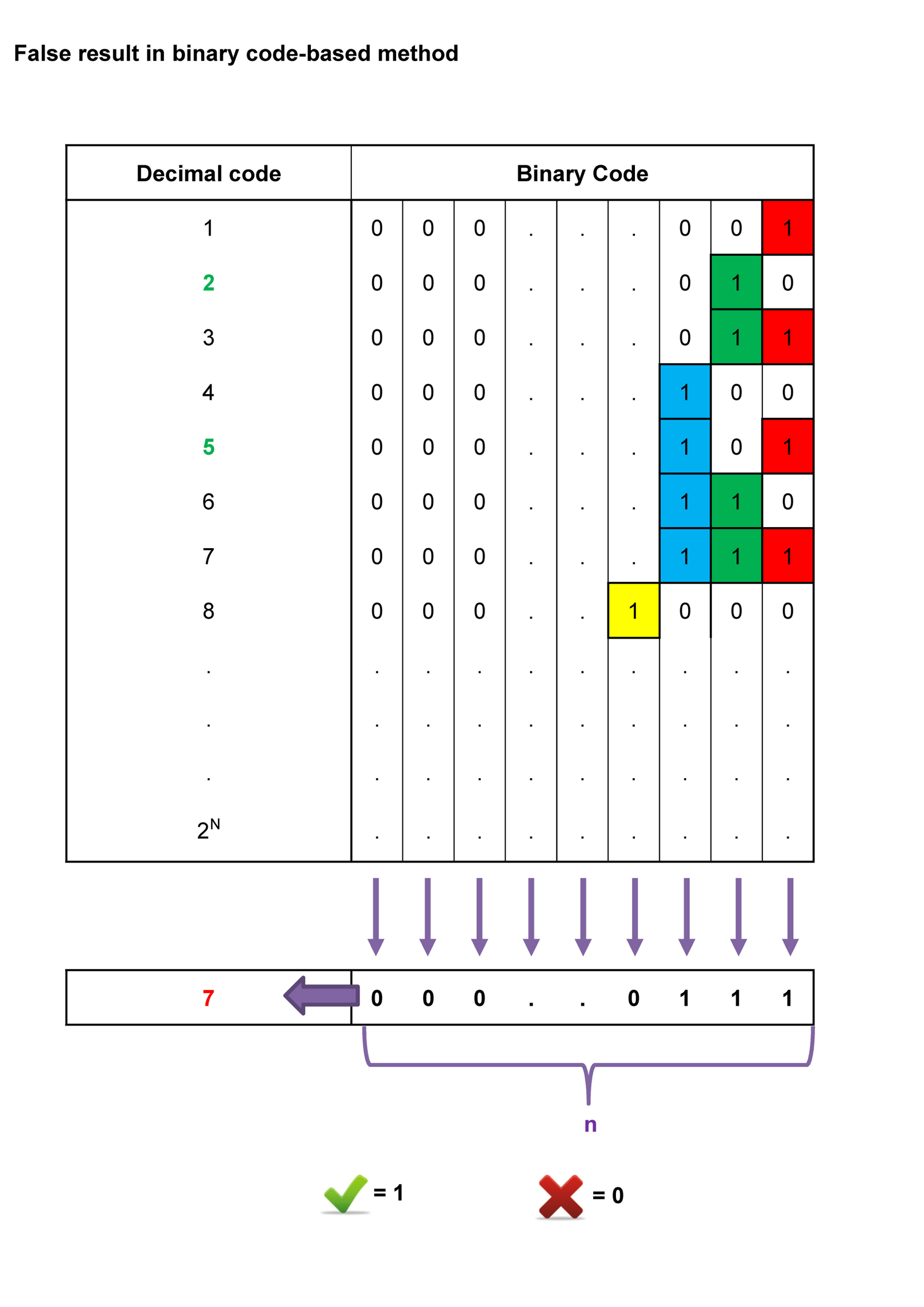

Supplement: S10 Fig — The binary-code method even produces a wrong result in this case (for example, if samples No. 2 and No. 5 are both positive samples, this method will give a wrong result: No. 7). (TIF) [file pone.0116997.s010.tif]
