# Supplementary material for: A Colony Multiplex Quantitative PCR-Based 3S3DBC Method and Variations of It for Screening DNA Libraries
Source: PLoS One. 2015 Feb 3;10(2):e0116997. doi: 10.1371/journal.pone.0116997 (PMC4315571; doi:10.1371/journal.pone.0116997)
Supplement: S1 Table — Positive and negative relationships of issues (x, y and z) that must be considered when designing a screening method are listed in the table. The functions relating x, y and z (z = f(y); y = f(x)), and their suitable solutions depend on different research laboratories’ particular situations. (DOCX) [file pone.0116997.s011.docx]

**Table S1.** Relationships among issues that must be considered when designing a screening method.

| Issues that must be considered during screening | | X | | | |
| --- | --- | --- | --- | --- | --- |
|  |  | Pooling density | | Multiplex primers | |
| y | Detection Number | - | | - | |
|  | Detection Step | - | | - | |
|  | **Detection Sensitivity** | - | | - | |
|  | **Detection Accuracy** | - | | - | |
|  | | | | | |
| Issues that must be considered during screening | | Y | | | |
|  |  | Number of PCR reactions | Number of PCR rounds | | Automation |
| z | **Time** | + | + | | - |
|  | **Labor** | + | + | | - |
|  | **Cost** | + | + | | - |

Positive and negative relationships of issues (x, y and z) that must be considered when designing a screening method are listed in the table. The functions relating x, y and z (z=f(y); y=f(x)), and their suitable solutions depend on different research labs’ particular situations.
