# Supplementary material for: A Colony Multiplex Quantitative PCR-Based 3S3DBC Method and Variations of It for Screening DNA Libraries
Source: PLoS One. 2015 Feb 3;10(2):e0116997. doi: 10.1371/journal.pone.0116997 (PMC4315571; doi:10.1371/journal.pone.0116997)
Supplement: S2 Table — (DOCX) [file pone.0116997.s012.docx]

**Table S2.** Comparison of screening one desired clone from DNA library by 3S3DBC screening method and conventional 3-dimensional method.

| Features | S3DBC screening method strategy in our actual case | Conventional PCR screening using 3D pooling strategy |
| --- | --- | --- |
| Maximum number of 384-well plates in one super pool | ~15 | ~10 |
| Number of PCR reactions needed to identify a positive super pool | n/15 | n/10 |
| Reactions needed to identify the plate ID for one positive super pool | 4 | 10 |
| Reactions needed to identify the clone ID from one positive plate | 10 | 40 |
| Total number of reactions needed to get positive BAC clone ID from whole library | n/15 + 14 | n/10 + 50 |
| Multiplexing possibility | Yes | No |
| Checking on agarose gel No | NO | Needed |
| Cost | ~ $0.34 per qPCR reaction | ~ $0.3 per PCR reaction + agarose gel electrophoresis |
| Procedure duration to screen out one desired clone | ~ 6 h | ~ 12 h |
| Data retrieved | Automatically reported from qPCR dissociation curve | Manually check from gel photos |

“n” is the total number of plates in the DNA library
